# Lipedema: Complications in High-Volume Liposuction Are Linked to Preoperative Anemia

**DOI:** 10.3390/jcm13247779

**Published:** 2024-12-19

**Authors:** Tonatiuh Flores, Barbara Kremsner, Jana Schön, Julia Riedl, Hugo Sabitzer, Christina Glisic, Kristina Pfoser, Jakob Nedomansky, Konstantin D. Bergmeister, Klaus F. Schrögendorfer

**Affiliations:** 1 Karl Landsteiner University of Health Sciences, Dr. Karl-Dorrek-Straße 30, 3500 Krems, Austria; barbara.kremsner@oegk.at (B.K.); jana.schoen@gmx.net (J.S.); hugo.sabitzer@stpoelten.lknoe.at (H.S.); christina.glisisc@stpoelten.lknoe.at (C.G.); kristina.pfoser@stpoelten.lknoe.at (K.P.); jakob.nedomansky@stpoelten.lknoe.at (J.N.); konstantin.bergmeister@stpoelten.lknoe.at (K.D.B.); klaus.schroegendorfer@stpoelten.lknoe.at (K.F.S.); 2 Clinical Department of Plastic, Aesthetic and Reconstructive Surgery, University Clinic of St. Poelten, 3100 St. Poelten, Austria; 3 Department of Medicine I, Division of Hematology and Hemostaseology, Medical University of Vienna, 1090 Vienna, Austria; julia.riedl@meduniwien.ac.at; 4 Clinical Laboratory for Bionic Extremity Reconstruction, University Clinic for Plastic, Reconstructive and Aesthetic Surgery, Medical University of Vienna, 1090 Vienna, Austria

**Keywords:** lipedema, hemoglobin loss, patient safety, high-volume liposuction

## Abstract

**Background:** Lipedema is a subcutaneous adipose tissue disorder mainly affecting women. Its progressive nature often requires high-volume liposuction for efficient pain reduction. However, aspiration volumes of more than 5 L within a single session may lead to a variety of complications. Thus, we examined the effect of high-volume liposuctions on lipedema patients and the incidence of associated complications. **Methods:** We analyzed perioperative differences in lipedema patients undergoing low- or high-volume liposuctions. Statistical analyses were performed, investigating postoperative complications and the correlation of patients’ BMI, total amount of aspiration, duration of surgery, hospital stay and hemoglobin alterations. Complications were investigated according to the Clavien–Dindo Classification. Patients were divided in two groups based on the volume aspirated at liposuction (low-volume vs. high-volume liposuction). **Results:** Overall, 121 sessions were investigated. Mean total volume of aspiration was 8227.851 mL ± 3643.891. Mean preoperative hemoglobin levels were 13.646 g/dL ± 1.075 g/dL. Preoperatively, 7.44% of patients were anemic (Hb < 12 g/dL). Mean postoperative hemoglobin was 10.563 g/dL ± 1.230 g/dL. Postoperatively, 90.10% of patients showed Hb levels below 12 g/dL. Hemoglobin loss differed significantly between the two groups (*p* = 0.001). Significant correlations between pre- (*p* = 0.015) and postoperative (*p* < 0.001) hemoglobin levels and pre- (*p* < 0.001) and postoperative (*p* < 0.001) anemia with Class II complications were also seen. The total volume of aspiration did not correlate with complication rates (*p* = 0.176). **Conclusions:** Complication rates in high-volume liposuctions are hemoglobin-dependent rather than volume-associated. Preoperative anemia was the most influential for the occurrence of postoperative complications. To safely conduct high-volume liposuctions in lipedema patients, adequate patient selection and preoperative patient preparation are imperative.

## 1. Introduction

Lipedema is a subcutaneous tissue disorder affecting adipocytes, predominantly encountered in women [1,2,3]. First described by Allen and Hines in 1940, it presents as a disproportionate accumulation of adipose tissue in upper and lower limbs. Pathological inflammation of subcutaneous tissue entails fibrotic alterations and pain, resulting in symmetrical swelling of the limbs, omitting the hands and feet [4,5,6,7]. Lipedema is often confounded in the case of obesity due to the increased body mass index (BMI) attributed to the presence of swollen extremities [2,8,9]. Additionally, it is resistant to modern diets and lifestyle changes [2,8,9,10,11,12].

Because of disease progression, many patients already suffer from advanced stages of lipedema, thus requiring high-volume liposuction (liposuction above 5 L of total aspirate per session) [7,13,14]. Thereby, lipedema reduction can be sufficiently addressed while conducting only few surgeries, even when upper and lower extremities are affected [15,16]. Yet, high-volume liposuctions may lead to various side-effects or complications [16,17]. Increased blood loss, Vitamin D depletion, or prolonged pain are encountered most frequently [7,16]. Thus, in this paper, we analyze high-volume liposuctions in lipedema patients on account of perioperative patient safety. We further aimed to disclose the feasibility of high-volume liposuction in lipedema patients. This research intends to support physicians safely performing high-volume liposuction in maximum-care facilities.

## 2. Materials and Methods

### 2.1. Study Design and Patient Analysis

In our study, we analyzed liposuctions of the lower extremities in lipedema patients between 1 January 2018 and 30 April 2021 at the Department of Plastic, Aesthetic and Reconstructive Surgery at the University Clinic of St. Poelten. The study was carried out as a retrospective, single-center study. Data were collected pseudonymously and adhered to Austrian data protection legislation. A standardized mean follow-up of one year was carried out at our department. Approval was granted by the ethics committee from the local institutional review board at the Karl Landsteiner University of Health Sciences Krems (reference number: ECS 1041/2021). The analyzed study data include the patient’s age, the duration of surgery, the patient’s BMI, the length of hospital stay, pre- and postoperative hemoglobin values (hb), dosage and duration of antithrombotic prophylaxis, infiltration volume and volume of aspiration during the liposuction. Further, we analyzed postoperatively encountered complications in concordance with the Clavien–Dindo Classification system. A 5-scale grading system was used to rank complications based on the therapy needed. It assists in stratifying the severity of complications, as a reliable and uniform tool. While Class III and IV entail surgical intervention, Class I and II only require pharmacological support (e.g., blood transfusions in Class II). Class V describes the patient’s death. Anemia was disclosed as values below 12 g/dL according to the WHO classification [18]. Adiposity was defined as a BMI > 30 kg/m^2^ according to the WHO classification [19].

### 2.2. Operative Procedure

In our institution, liposuction is performed under general anesthesia with the tumescence technique. Preoperatively, patients are examined and the areas requiring treatment are marked while standing for accurate identification. Antibiotic prophylaxis is administrated at least 30 min before the primary surgical incision and continued for one week postoperatively. Patients either receive 2.2 g of amoxicillin/clavulanic acid (Curam^®^, Sandoz GmbH, 6250 Kundl, Austria) or 600 mg of clindamycin (Dalacin^®^, Fareva Amboise Zone Industrielle, Routes des Industries 29, 37530 Pocé-sur-Cisse, France) in case of a penicillin allergy. For liposuction, we install a modified Klein’s solution with 1000 mL Ringer’s lactate (Ringer lactate^®^, Fresenius Kabi, Rue du Rempart 6, 27400 Louviers, France) containing one milliliter of 1:1000 epinephrin (Suprarenin^®^ Sanofi-Aventis GmbH, 65926 Frankfurt am Main, Germany). The solution is infiltrated through small stab incisions placed at strategically selected locations by using a number eleven blade. These incisions are placed in areas easy to conceal postoperatively, e.g., by the patients clothing. After fifteen minutes of indwelling time for the tumescent solution to set, vibration-assisted liposuction (VAL) is performed using 3 and/or 4 mm multiport cannulas (multiport rapid extraction cannula, Moeller Medical^®^ GmbH, Wasserkuppenstraße 29–31, 36043 Fulda, Germany) paired with a Moeller’s liposuction device (Moeller Vibrasat Pro, Moeller medical^®^ GmbH, Wasserkuppenstraße 29–31, 36043 Fulda, Germany).

After liposuction, the incisions are rinsed with Octenisept^®^ (Schülke & Mayr GmbH, Robert-Koch-Straße 2, 22851, Norderstedt, Germany) and Skinsept^®^ (Ecolab Germany GmbH, Ecolab-Allee 1, 40789 Monheim am Rhein, Germany) followed by plaster coating. Stab incisions are not sutured at our department, to allow for the tumescence solution to drain. Compression garments are applied while the patient is still in the operating room. Compression must be worn continuously, day and night, for the following three months. Patients receive antibiotic shielding for an additional seven days postoperatively and antithrombotic prophylaxis using low molecular heparin for 10 to 30 days postoperatively. Patients additionally receive 500–1500 mL saline solution by default at the ward.

### 2.3. Statistical Analyses

All collected patient data in the selected timeframe were pseudonymized. Data protection management was performed adhering to Austrian legislation. The collection and processing of the necessary patient information was carried out using Microsoft Excel (version 2010, Microsoft, Redmond, WA, USA, Version 4.2.0 for Windows (22 April 2022). Statistical analyses were conducted with IBM SPSS Statistics for Windows Version (Version 29, IBM, Armonk, NY, USA). Nominal data were described with absolute frequencies and percentages, while metric data were summarized using means and standard deviations. Further analyses investigating the impact of complications on postoperative patient recovery, *t*-tests for independent samples, and Spearman-Rho correlation analyses were performed. Results were considered significant in the case of *p* < 0.05.

## 3. Results

### 3.1. Demographics

Within our study we analyzed 184 liposuction sessions in 107 patients suffering from lipedema. Here, 38 liposuctions were excluded, as they were performed on the upper extremities, so as to not distort the dataset and to accurately compare our groups. Additionally, 25 sessions were excluded due to a lack of data (postoperative hemoglobin levels acquired after 48 h of surgery). Finally, 121 liposuctions in 90 patients met our criteria and were included in this study. Our dataset included exclusively women. Patients were further divided upon receiving low-volume liposuction (total volume aspirated ≤ 5 L) or high-volume liposuctions (total volume aspirated > 5 L). In total, 25 (20.66%) sessions of low-volume liposuction were performed, and 96 (79.34%) of high-volume liposuction.

Patients mean age was 39.969 years ± 12.244 years (Table 1). Mean overall BMI was 32.013 kg/m^2^ ± 7.135 kg/m^2^. Overall, 81 (66.94%) patients showed a BMI > 30 kg/m^2^. Hospital stay was on average 4.27 days ± 1.08 days. The mean duration of surgery was 112.363 min ± 27.877 min. The total amount of infiltration was 7363.636 mL ± 2423.633 mL. The mean total volume of aspiration was 8227.851 mL ± 3643.891 mL. The mean preoperative hemoglobin level was 13.646 g/dL ± 1.075 g/dL. Postoperative hemoglobin had a mean of mean 10.563 g/dL ± 1.230 g/dL. The mean hemoglobin loss was 3.052 g/dL ± 1.191 g/dL. The duration of antithrombotic prophylaxis using low-molecular heparin had a mean of 18.50 days ± 11.51 days. Antithrombotic dosages had a mean of 43.801 mL ± 8.684 mL.

In total, 20 (16.52%) sessions were associated with postoperative complications adhering to the Clavien–Dindo Classification. Hereby, 2 (1.65%) were seen in patients experiencing low-volume liposuction (≤5 L aspirate) and 18 (14.88%) were seen in women experiencing high-volume liposuction (>5 L aspirate).

#### 3.1.1. Low-Volume Liposuction

In total, 25 liposuctions were performed with an aspiration volume of less than or equal to 5 L. The mean age was 42.52 years ± 12.965 years. The mean duration of surgery in this group was 94.920 min ± 31.303 min. Total infiltration had a mean of 4952.00 mL ± 1366.296 mL. The mean volume of aspiration was 3844.800 ± 897.339 mL. Hospital stay had a mean of 4.00 days ± 1.000 day in this group. BMI had a mean of 28.304 kg/m^2^ ± 5.523 kg/m^2^. In this group, 15 patients (60%) had BMI > 25 kg/m^2^. Preoperative hemoglobin was 13.296 g/dL ± 1.060 g/dL. Here, four (16%) women showed preoperative anemia. Postoperative hemoglobin was 10.924 g/dL ± 1.109 g/dL, with 22 (88%) patients experiencing postoperative anemia. Antithrombotic prophylaxis was given on mean for 11.20 days ± 5.97 days. The mean low-molecular heparin dosage was 40.000 mL.

Postoperative complications in low-volume patients were solely Class I. The main complications were slight dizziness and emesis, which required the use of antiemetics, presumably as a consequence of anesthesia. All complications ceased within 12 h and were not present at time of discharge.

#### 3.1.2. High-Volume Liposuction

In this group, 96 women experienced liposuction above 5 L in total. The mean patient age was 39.30 years ± 12.031 years. The duration of surgery was 116.906 min ± 25.162 min on average. The total infiltration had a mean of 7991.666 mL ± 2240.332 mL. The mean volume of aspiration was 9369.270 ± 3193.211 mL. Hospital stay length had a mean of 4.35 days ± 1.10 days in this group. The mean BMI was 32.978 kg/m^2^ ± 7.213 kg/m^2^. Women experiencing high-volume liposuction showed a BMI > 25 kg/m^2^ in 83 (86.46%) cases. Preoperative hemoglobin was 13.700 g/dL ± 1.069 g/dL. Here, five (5,21%) patients had preoperative anemia. Postoperative hemoglobin was 10.469 g/dL ± 1.247 g/dL, with 87 (90.63%) women experiencing postoperative anemia. Antithrombotic prophylaxis was obtained based on the mean of 20.41 days ± 11.86 days. The mean low-molecular heparin dosage was 44.791 mL ± 9.512 mL.

High-volume liposuction patients experienced 14 (11.57%) Class I complications, involving requiring only antiemetics or electrolytes. All Class I complications ceased after 12 h and where not present at time of discharge. The remaining four (3.30%) patients experienced Class II complications. Here, all patients received one blood transfusion postoperatively due to hemoglobin drop below 7.6 g/dL and hemodynamic manifestation. Two of the patients receiving blood transfusion had a history of varicose.

### 3.2. Statistical Analyses

#### 3.2.1. Volume-Associated Statistical Analyses

**Figure 1:**
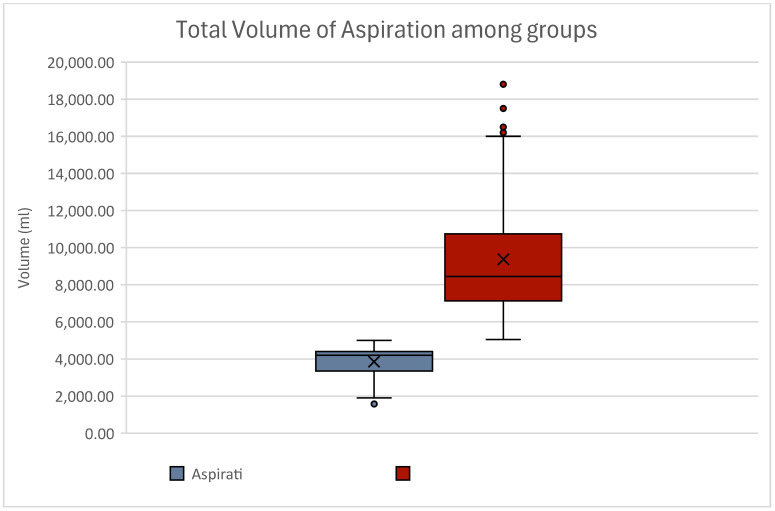
Boxplot of the total volume of aspiration between groups. As women were grouped into low-volume (≤5 L total aspiration) and high-volume (>5 L total aspiration) liposuction, minimum, maximum and mean values differed significantly (*p* < 0.01). Although statistically significant, these results show no clinical relevance. Women experiencing low-volume liposuction are displayed in blue; women experiencing high-volume liposuction are displayed in red. Outliers can be seen as dots.

Although our groups were unevenly distributed, statistical analyses were feasible. Statistical results proved to be significant, as sufficient data were present to entail reliable statistical power. Thus, a power analysis was not necessary. Due to the division of our patients into low-volume and high-volume liposuction, statistical analyses involving *t*-tests of independent samples showed obvious significances when analyzing liposuction volumes. The total volume of infiltration and aspiration differed significantly (*p* < 0.01) (Figure 1).

#### 3.2.2. Surgery-Associated Statistical Analyses

**Figure 2:**
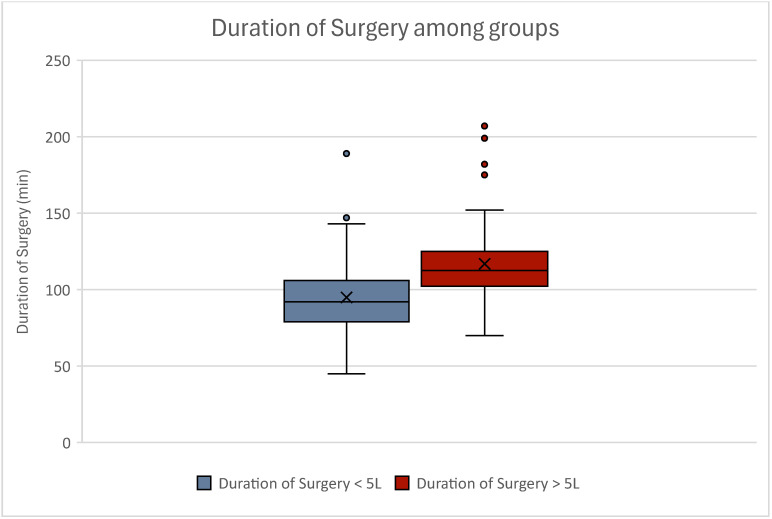
Boxplot of duration of surgery among groups. Women experiencing high-volume liposuction had significantly higher duration of surgery (*p* < 0.001). Women experiencing low-volume liposuction are displayed in blue; women experiencing high-volume liposuction are displayed in red. Outliers can be seen as dots.

Comparing the duration of surgery between our groups, a statistical significance could be observed. Women experiencing high-volume liposuction showed significantly higher surgery time than women experiencing low-volume liposuction (*p* < 0.001) (Table 2). Although this finding was expected, the means of the duration of surgery between our groups are not as far apart as anticipated (Figure 2).

**Figure 3:**
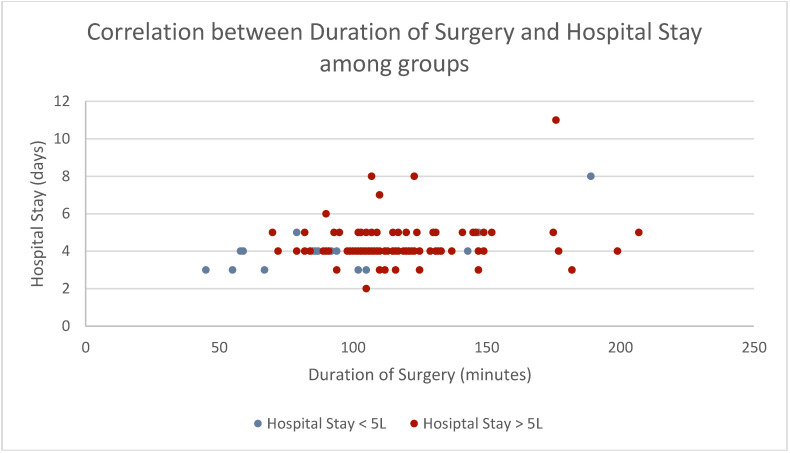
Scatter plot correlating duration of surgery to hospital stay, showing an extension in hospital stay with longer surgery times. A significant correlation can be observed (*p* = 0.019). Women experiencing low-volume liposuction are displayed in blue; women experiencing high-volume liposuction are displayed in red.

No statistical significance between in-hospital durations (*p* = 0.160) was observed. Yet, when performing Spearman Rho analyses to correlate duration of surgery to hospital stay, we observed a statistical significance (*p* = 0.019) demonstrating that a longer duration of surgery significantly extends hospital stay (Table 3, Figure 3).

Further, higher total aspiration volumes directly extend hospital stay (*p* < 0.001) (Table 4).

#### 3.2.3. Patient-Associated Statistical Analyses

**Figure 4:**
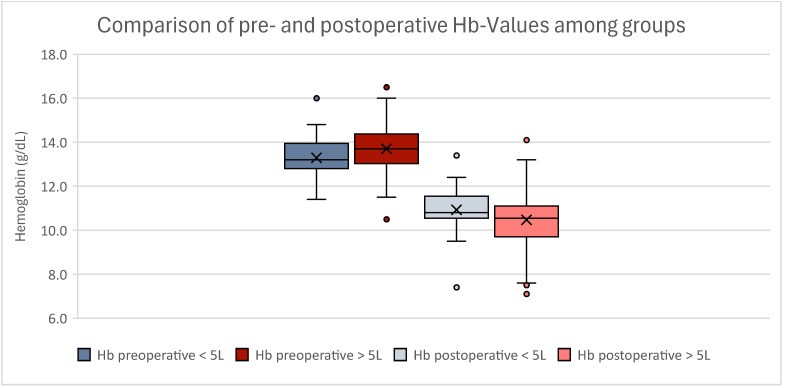
Boxplot of pre- and postoperative hemoglobin values. *t*-test analyses showed no statistical significance within either group. Women experiencing low-volume liposuction are displayed in blue; women experiencing high-volume liposuction are displayed in red. Outliers can be seen as dots.

Conducting *t*-test analyses, no significant difference regarding BMI (*p* = 0.03) and age (*p* = 0.243) between our groups was seen. Our analyses of preoperative hemoglobin levels also showed no significance (*p* = 0.094). Postoperative hemoglobin values did not differ significantly either (*p* = 0.100) (Figure 4). There was also no significant difference in preoperative (*p* = 0.178) or postoperative anemia (*p* = 0.699) between low- and high-volume liposuction patients.

**Figure 5:**
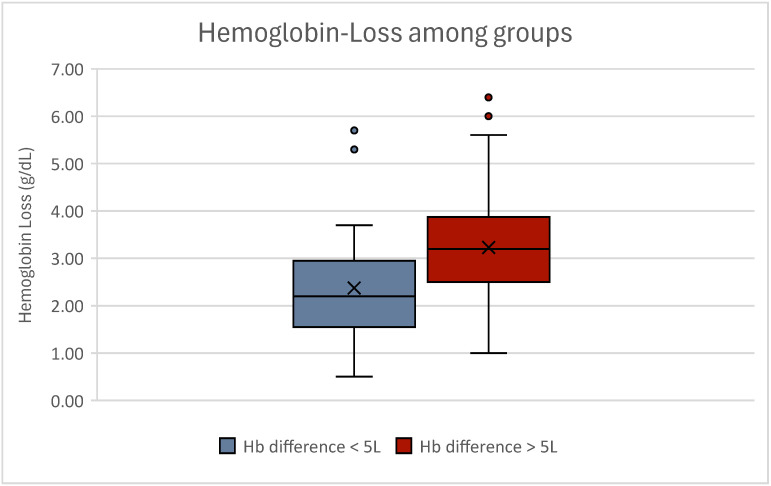
Boxplot of hemoglobin loss between both groups. Statistical analyses showed a significant difference regarding hemoglobin loss (*p* = 0.001). Women experiencing low-volume liposuction are displayed in blue; women experiencing high-volume liposuction are displayed in red. Outliers can be seen as dots.

Yet, hemoglobin loss between low- and high-volume liposuction patients did differ significantly (*p* = 0.001), demonstrating that high-volume liposuction sessions entail higher blood-loss than low-volume liposuction sessions (Table 5, Figure 5).

**Figure 6:**
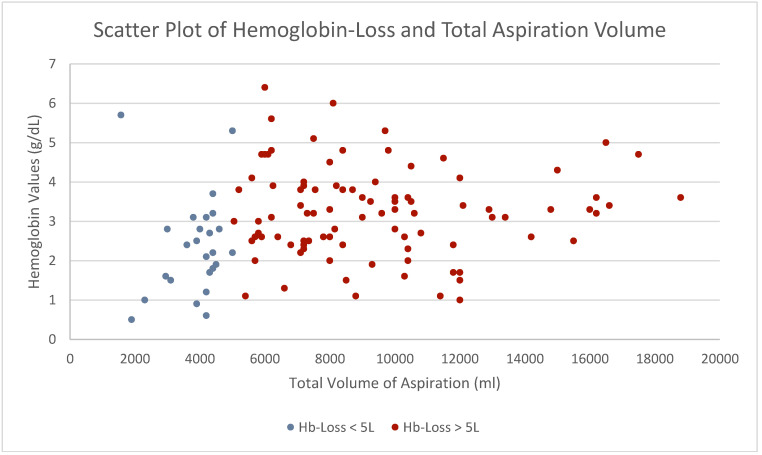
Scatter plot correlating hemoglobin loss to total volume of aspiration. A direct correlation can be seen within our study (*p* = 0.012). Women experiencing low-volume liposuction are displayed in blue; women experiencing high-volume liposuction are displayed in red.

Comparing both groups, no significance in postoperative complication rate adhering to the Clavien–Dindo Classification could be seen (*p* = 0.122). Performing analyses correlating hemoglobin loss to total aspiration volume, we observed a significant correlation (*p* = 0.012) (Table 6, Figure 6).

BMI and duration of surgery still did not correlate significantly to hemoglobin loss (p_BMI_ = 0.620, p_duration_ = 0.454).

Investigating complication rates using the Clavien–Dindo Scale, no significant correlation could be seen regarding the duration of surgery (*p* = 0.101), age (*p* = 0.116), or BMI (*p* = 0.143). Also, no significant correlation could be seen between complication rates and the total volume of aspiration (0.176) (Table 7).

Nonetheless, a statistical significance was seen between complication rates and hemoglobin loss (*p* = 0.002) (Table 8).

Postoperatively, 99 (81.82%) patients showed hemoglobin levels below 12 g/dL. In total, 101 (83.47%) patients did not show any kind of postoperative issues, while 20 (16.53%) patients did show complications, based on the Clavien–Dindo Classification. When performing independent *t*-tests, we observed a significant difference in hospital stay (*p* = 0.012) and duration of postoperative antithrombotic prophylaxis (*p* = 0.05) between patients with and without postoperative complications.

When comparing hemoglobin values and anemia, we also encountered statistical significances. Postoperative hemoglobin levels differed significantly between patients without and with postoperative complications (*p* < 0.001). Yet, preoperative hemoglobin values did not differ significantly (*p* = 0.080). Still, a tendency can be seen. The occurrence of anemia was additionally seen to be significantly different, both pre- (*p* = 0.050) and postoperatively (*p* < 0.001) (Table 9).

Conducting Spearman Rho correlation analyses, we additionally deciphered complications significantly correlating with hemoglobin values. Pre- (*p* = 0.015) and postoperative (*p* <0.001) hemoglobin levels significantly correlated with the occurrence of postoperative complications. This was also true for hemoglobin loss (*p* = 0.001) and even the percentual difference in postoperative hemoglobin loss (*p* < 0.001). Further, preoperative anemia also correlated significantly with postoperative complication rates (*p* < 0.001). Still, postoperative anemia did not correlate significantly to higher complication rates (*p* = 0.053) (Table 10).

## 4. Discussion

Lipedema is an adipose tissue disorder mainly affecting women [10]. Since many patients show elevated BMI, high-volume reduction is often necessary [7,17]. This can either be achieved by performing several sessions of liposuction, or through less high-volume liposuction. Yet, multiple procedures entail higher costs for health care systems and patients, and require multiple applications of anesthesia, while only providing slow symptom relief [20]. Thus, high-volume liposuctions are more efficient and more patient-adapted [21,22,23]. Unfortunately, high-volume liposuctions have been condemned as risky procedures, as complication rates seem to increase with volume aspirated [24,25,26]. Nonetheless, liposuctions above this threshold are widely performed nowadays [16].

In our cohort, the mean total volume of aspiration was 8227.851 mL ± 3643.891 mL with a maximum of 18,800 mL in one session. However, our number of Class II complications of 3.31% did not exceed common rates described in the literature with a lower total volume aspirated [24]. Further, we did not see any significant difference in hospital stay, postoperative hemoglobin levels or overall complication rates between low- and high-volume liposuction sessions. Naturally, a significant difference in the duration of surgery and total volume of aspiration was apparent. Yet, these findings hold no clinical relevance, as a higher volume of aspiration requires more surgery time. Also, because our patients were divided into low- and high-volume liposuction groups, this result was expected. Still, the observed gap between the respective mean durations of surgery between our groups was not as significant as expected.

Among our patients, hemoglobin loss differed significantly (*p* = 0.001). Additionally, hemoglobin loss directly correlated with total volume of aspiration (*p* = 0.012). Interestingly, the total volume of aspiration did not correlate with complication rates (*p* = 0.176). As such, we assume that adverse events in high-volume liposuction are hemoglobin-dependent rather than volume-affiliated. Naturally, hemoglobin loss correlated directly with Class II complications (*p* = 0.002); nonetheless, this result was to be expected, as all patients experiencing Class II complications received blood transfusions.

These findings were also supported by the significant correlation between preoperative anemia and the presence of complications (*p* < 0.001), and the fact that women experiencing Class II complications showed preoperative anemia in 75% of cases. When stratifying patients based on the presence or absence of postoperative complications, those who experienced complications exhibited significantly lower perioperative hemoglobin levels compared to those without complications. Additionally, the incidence of postoperative anemia differed significantly between the groups. Correlating complication rates with hematologic parameters, we revealed significant associations with preoperative low hemoglobin levels (*p* = 0.015) and preoperative anemia (*p* < 0.001), but not with postoperative anemia (*p* = 0.053). These findings underscore the critical role of preoperative anemia as a pivotal factor in the development of postoperative complications. Finally, a direct correlation between complications and the duration of surgery could not be confirmed (*p* = 0.101). Neither could a longer duration of surgery be correlated to hemoglobin loss (*p* = 0.454).

Further, we identified preoperative anemia as a direct risk factor for postoperative complications in high-volume liposuction. This recognition was also seen in, e.g., anemic breast cancer patients experiencing higher drainage fluid volume after mastectomy [18], thus highlighting the key role of hemoglobin and its significance in postoperative sequelae. Therefore, lipedema patients should be evaluated precisely before surgery. As for the selective nature of high-volume liposuction, preoperative preparation is fundamental for patient safety [27].

Unfortunately, our study faces some limitations. Our department’s focus on efficiently minimizing patients’ pain may introduce a bias favoring high-volume liposuction. Further, the uneven distribution between groups poses a risk of distorting statistical analyses. Yet, as for the progressive nature of this disease, low-volume liposuctions are only seldomly encountered in our department. To more precisely address this important topic, prospective multicenter studies ought to be conducted, whereby the uneven patient distribution could be reduced. Further, long-term follow-ups need to be emphasized, to eradicate disruptive factors such as the under-reporting of possible complications after hospital discharge. Although none of our patients related such information, precise questioning could be implemented. Another limiting aspect could be the modified Klein’s solution. The adding of adrenaline to our tumescence solution is intended to achieve vasoconstriction, which reduces bleeding and minimizes hemoglobin loss. Furthermore, the use of larger volumes of tumescence solution in high-volume liposuction may amplify this effect. Finally, the employment of bigger cannulas might also cause increased bleeding.

## 5. Conclusions

Our study highlights the significance of hemoglobin-dependency in complication rates of high-volume liposuction. Preoperatively anemic patients planned for high-volume liposuctions should be reconsidered or sufficiently prepared. Additionally, hematologic characteristics ought to be optimized, ahead of high-volume liposuction. By sustaining these features in high-volume liposuction, along with general perioperative security guidelines, this procedure can be improved upon in terms of feasibility and patient safety.

## Figures and Tables

**Table 1:** Demography of patients included in this study.

Patient Characteristics		Lipoaspirate < 5 L	Lipoaspirate > 5 L	Total
Number		25 (20.66%)	96 (79.34%)	121
Age (years)	Mean	42.52	39.30	39.969
	Min–Max	23–66	19–72	19–72
	STD	±12.965	±12.031	±12.244
BMI (kg/m^2^)	Mean	28.304	32.978	32.013
	Min–Max	19.7–37.9	21–58.40	19.70–58.40
	STD	±5.523	±7.213	±7.135
Duration of Surgery (min)	Mean	94.920	116.906	112.363
	Min–Max	45–189	70–207	45–207
	STD	±31.303	±25.162	±27.877
Hospital Stay (days)	Mean	4.000	4.343	4.272
	Min–Max	3–8	2–11	2.0–11
	STD	±1.000	±1.103	±1.087
Volume of Infiltration (mL)	Mean	4952.000	7991.666	7363.636
	Min–Max	1100–6000	2000–14,000	2000.00–14,000.00
	STD	±1366.296	±2240.332	±2423.633
Volume of Aspiration (mL)	Mean	3844.800	9369.270	8227.851
	Min–Max	1570–5000	5050–18,800	1570.00–18,800.00
	STD	±897.339	±3193.211	±3643.891
Hemoglobin, preoperative (g/dL)	Mean	13.296	13.700	13.646
	Min–Max	11.5–16.0	10.5–16.5	10.5–16.5
	STD	±1.060	±1.069	±1.075
Hemoglobin, postoperative (g/dL)	Mean	10.924	10.469	10.563
	Min–Max	7.4–13.4	7.1–14.1	7.1–14.1
	STD	±1.109	±1.247	±1.230
Hemoglobin Loss (g/dL)	Mean	2.372	3.230	3.052
	Min–Max	0.5–5.7	1.1–6.4	0.5–6.4
	STD	±1.264	±1.111	±1.191
Anemia, preoperative	N	4 (16%)	5 (5.21%)	9
Anemia, postoperative	N	22 (88%)	87 (90.63%)	109
Antithrombosis prophylaxis dosage (mL)	Mean	40.000	44.791	43.801
	Min–Max	40–40	40–80	40–80
	STD	±0.000	±9.512	±8.684
Antithrombosis prophylaxis duration (days)	Mean	11.200	20.406	18.504
	Min–Max	5–14	6–56	5–56
	STD	5.972	±11.858	±11.509

**Table 2:** Levene’s Test analyzing duration of surgery between groups. Our analyses showed a significant difference in the duration of surgery between low- and high-volume liposuction (*p* < 0.001).

Levene’s Test of Equality of Variances							
	F	Sig.	T	df	One-sided *p*	Two-sided *p*	Mean difference
Duration of Surgery	0.818	0.368	−3.693	119	<0.001	<0.001	−21.986

**Table 3:** Spearman Rho Rank analysis showing that duration of surgery has a significant impact on hospital stay (*p* = 0.019).

Spearman Rho Correlation Analysis				
			Duration of Surgery	Hospital Stay
Spearman Rho	Duration of Surgery	Corr. Coefficient	1.000	0.213
		Sig. (2-tailed)		0.019
		N	121	121
	Hospital Stay	Corr. Coefficient	0.213	1.000
		Sig. (2-tailed)	0.019	
		N	121	121

**Table 4:** Spearman Rho Rank analysis showing that total volume of aspiration has a significant impact on hospital stay (*p* < 0.001).

Spearman Rho Correlation Analysis				
			Volume total Aspiration	Hospital Stay
Spearman Rho	Volume total Aspiration	Corr. Coefficient	1.000	0.335
		Sig. (2-tailed)		<0.001
		N	121	121
	Hospital Stay	Corr. Coefficient	0.335	1.000
		Sig. (2-tailed)	<0.001	
		N	121	121

**Table 5:** Levene’s Test showing a statistical significance regarding hemoglobin loss between both groups (*p* < 0.001).

Levene’s Test of Equality of Variances							
	F	Sig.	T	df	One-sided *p*	Two-sided *p*	Mean difference
Hemoglobin Loss	0.166	0.685	−3.341	119	<0.001	0.001	−0.858

**Table 6:** Spearman Rho Rank analysis showing that total volume of aspiration has a significant impact on hemoglobin loss (*p* = 0.012).

Spearman Rho Correlation Analysis				
			Volume total Aspiration	Hemoglobin Loss
Spearman Rho	Volume total Aspiration	Corr. Coefficient	1.000	0.227
		Sig. (2-tailed)		0.012
		N	121	121
	Hemoglobin Loss	Corr. Coefficient	0.227	1.000
		Sig. (2-tailed)	0.012	
		N	121	121

**Table 7:** Spearman Rho Rank analysis showing that the total volume of aspiration and complication rates do not correlate (*p* = 0.176).

Spearman Rho Correlation Analysis				
			Complication Rates	Volume total Aspiration
Spearman Rho	Complication rates	Corr. Coefficient	1.000	0.124
		Sig. (2-tailed)		0.176
		N	121	121
	Volume total Aspiration	Corr. Coefficient	0.124	1.000
		Sig. (2-tailed)	0.176	
		N	121	121

**Table 8:** Spearman Rho Rank analysis showing that postoperative complication rates significantly correlate to hemoglobin loss (*p* = 0.002).

Spearman Rho Correlation Analysis				
			Complication rates	Hemoglobin Loss
Spearman Rho	Complication rates	Corr. Coefficient	1.000	0.275
		Sig. (2-tailed)		0.002
		N	121	121
	Hemoglobin Loss	Corr. Coefficient	0.275	1.000
		Sig. (2-tailed)	0.002	
		N	121	121

**Table 9:** Levene’s Test of pre- and postoperative hemoglobin values and anemia compared between patients without and patients with postoperative complications. Here, a significant difference (*p* < 0.001) in postoperative hemoglobin values can be seen. Also, the occurrence of anemia, pre- (*p* = 0.050) and postoperatively (*p* < 0.001), showed statistical significance. Solely preoperative hemoglobin levels did not differ significantly (*p* = 0.080).

Levene’s Test of Equality of Variances							
	F	Sig.	T	df	One-sided *p*	Two-sided *p*	Mean difference
Hemoglobin preop	5.133	0.025	1.834	22.564	0.040	0.080	0.6128
Hemoglobin postop	3.463	0.065	5.195	119	<0.001	<0.001	1.4180
Anemia preop	42.321	<0.001	−2.078	20.487	0.025	0.050	−0.2104
Anemia postop	14.172	<0.001	−3.672	100	<0.001	<0.001	−0.1188

**Table 10:** Spearman Rho Rank analysis of complication rates in correlation to hemoglobin values preoperatively and postoperatively (p_preoperative_ = 0.015 and p_postoperative_ < 0.001), and anemia preoperatively and postoperatively (p_preoperative_ = y 0.001 and p_postoperative_ = 0.053).

Spearman Rho Correlation Analysis			
			Complication rates
Spearman Rho	Complication rates	Corr. Coefficient	1.000
		Sig. (2-tailed)	
		N	121
	Hemoglobin preop	Corr. Coefficient	−0.196
		Sig. (2-tailed)	0.015
		N	121
	Hemoglobin postop	Corr. Coefficient	−0.366
		Sig. (2-tailed)	<0.001
		N	121
	Anemia preop	Corr. Coefficient	0.298
		Sig. (2-tailed)	<0.001
		N	121
	Anemia postop	Corr. Coefficient	0.148
		Sig. (2-tailed)	0.053
		N	121

## Data Availability

All the data analyzed during the current study are available from the corresponding author on reasonable request.
